# Supplementary material for: Impact of Transcriptome and Gut Microbiome on the Response of HIV-1 Infected Individuals to a Dendritic Cell-Based HIV Therapeutic Vaccine
Source: Vaccines (Basel). 2021 Jun 24;9(7):694. doi: 10.3390/vaccines9070694 (PMC8310021; doi:10.3390/vaccines9070694)
Supplement: Supplementary file 1 [file vaccines-09-00694-s001.zip › vaccines-1237125-supplementary.pdf]

Supplementary Material

Table S1. Transcriptomics analysis of the mRNA from PBMCs in subjects who received the vaccine between the two time-points. The differential expressed genes (DE) correspond to non-coding RNA and did not presented a known function. It was presented the first 100 genes of the obtained list after the analysis.

ENSEMBL	Gene symbol	Pre-vaccination mean	Post-vaccination mean	PP	DE
**ENSG00000230629.2_RPS23P8**	RPS23P8	0.24	54.42	1.00	yes
**ENSG00000253861.1_SLC2A3P1**	SLC2A3P1	8.13	0.00	1.00	yes
**ENSG00000269968.1_RP5-940J5.9**	RP5-940J5.9	22.42	0.00	1.00	yes
**ENSG00000278774.1_U2**	U2	0.00	8.59	1.00	yes
**ENSG00000262413.1_RP11-498C9.3**	RP11-498C9.3	0.00	2.79	1.00	yes
**ENSG00000280496.1_SNORA43**	SNORA43	71.41	2.61	1.00	yes
**ENSG00000268220.1_RP11-379K17.12**	RP11-379K17.12	0.40	9.75	1.00	yes
**ENSG00000281220.1_AC011404.1**	AC011404.1	22.48	0.71	1.00	yes
**ENSG00000219928.2_RP11-40C6.2**	RP11-40C6.2	1.63	20.08	1.00	yes
**ENSG00000196656.7_AC004057.1**	AC004057.1	0.43	9.28	1.00	yes
**ENSG00000185641.6_CTD-2287O16.1**	CTD-2287O16.1	3.46	25.00	1.00	yes
**ENSG00000253683.1_CTB-79E8.3**	CTB-79E8.3	0.27	7.83	1.00	yes
**ENSG00000234851.4_RPL23AP42**	RPL23AP42	10.95	121.48	1.00	yes
**ENSG00000260349.1_RP11-473I1.5**	RP11-473I1.5	1.76	10.72	1.00	yes
**ENSG00000257767.2_RP11-162P23.2**	RP11-162P23.2	10.99	2.50	0.99	yes
**ENSG00000215837.7_SDHAP2**	SDHAP2	1.91	23.26	0.99	yes
**ENSG00000274049.4_INO80B-WBP1**	INO80B-WBP1	1.33	5.67	0.96	yes
**ENSG00000226976.3_COX6A1P2**	COX6A1P2	1.85	11.32	0.91	no
**ENSG00000259053.1_RP11-33N16.3**	RP11-33N16.3	0.13	2.43	0.90	no
**ENSG00000196205.8_EEF1A1P5**	EEF1A1P5	387.41	2352.85	0.87	no
**ENSG00000280614.1_CH507-513H4.4**	CH507-513H4.4	6479.37	27677.23	0.79	no
**ENSG00000280800.1_CH507-513H4.6**	CH507-513H4.6	6479.37	27677.23	0.79	no
**ENSG00000281181.1_CH507-513H4.3**	CH507-513H4.3	6479.37	27677.23	0.79	no
**ENSG00000265967.1_AC100830.4**	AC100830.4	2.38	0.14	0.77	no
**ENSG00000158497.2_HMHB1**	HMHB1	0.28	2.37	0.61	no
**ENSG00000211968.3_IGHV1-58**	IGHV1-58	3.96	0.58	0.58	no
**ENSG00000256034.1_RP11-707G14.1**	RP11-707G14.1	3.29	0.15	0.58	no
**ENSG00000253722.1_RP11-10N23.4**	RP11-10N23.4	1.17	3.78	0.56	no
**ENSG00000272991.1_AF129408.17**	AF129408.17	0.55	2.74	0.53	no
**ENSG00000225840.2_AC010970.2**	AC010970.2	2812.14	10193.96	0.46	no
**ENSG00000213300.5_HNRNPA3P6**	HNRNPA3P6	3.79	15.92	0.43	no
**ENSG00000251039.2_IGKV2D-40**	IGKV2D-40	2.16	0.16	0.40	no
**ENSG00000166670.9_MMP10**	MMP10	1.41	9.73	0.39	no
**ENSG00000282639.1_CH17-224D4.1**	CH17-224D4.1	9.41	1.80	0.38	no
**ENSG00000281383.1_CH507-513H4.5**	CH507-513H4.5	16.68	44.43	0.34	no
**ENSG00000281955.1_RP11-126O22.9**	RP11-126O22.9	0.04	0.85	0.28	no
**ENSG00000280959.1_ACEA_U3**	ACEA	0.15	2.05	0.27	no
**ENSG00000259132.1_RP11-298I3.5**	RP11-298I3.5	14.72	3.89	0.25	no
**ENSG00000262160.1_RP11-96D1.11**	RP11-96D1.11	1.83	4.69	0.24	no
**ENSG00000252106.2_RNY3P15**	RNY3P15	1.23	0.02	0.22	no
**ENSG00000274403.1_CTD-2036P10.6**	CTD-2036P10.6	13.92	21.06	0.21	no
**ENSG00000229344.1_RP5-857K21.7**	RP5-857K21.7	47.04	32.50	0.17	no
**ENSG00000235558.3_RP11-1198D22.1**	RP11-1198D22.1	0.00	3.07	0.15	no
**ENSG00000144820.7_ADGRG7**	ADGRG7	30.50	21.88	0.14	no
**ENSG00000241697.4_TMEFF1**	TMEFF1	1.15	0.00	0.14	no
**ENSG00000259546.1_RP11-351M8.2**	RP11-351M8.2	2.58	0.67	0.13	no
**ENSG00000281371.1_INE2**	INE2	1.31	6.78	0.12	no
**ENSG00000258017.1_RP11-386G11.10**	RP11-386G11.10	17.95	71.54	0.12	no

**ENSG00000223544.1_AC005838.2**	AC005838.2	1.45	0.13	0.11	no
**ENSG00000275203.1_AL583842.2**	AL583842.2	1.62	5.60	0.11	no
**ENSG00000235486.1_AC114755.7**	AC114755.7	1.16	0.00	0.10	no
**ENSG00000274582.1_SNORA16A**	SNORA16A	29.75	4.92	0.09	no
**ENSG00000262429.1_RP5-1050D4.3**	RP5-1050D4.3	0.04	0.81	0.09	no
**ENSG00000234793.1_AC114730.7**	AC114730.7	1.87	0.09	0.08	no
**ENSG00000233084.2_RPL23AP25**	RPL23AP25	4.02	1.30	0.08	no
**ENSG00000211938.2_IGHV3-7**	IGHV3-7	29.90	7.74	0.06	no
**ENSG00000275727.1_AC092375.1**	AC092375.1	9.21	3.62	0.06	no
**ENSG00000270075.1_RP11-127L20.5**	RP11-127L20.5	0.10	1.36	0.06	no
**ENSG00000273834.1_MIR612**	MIR612	2.70	8.78	0.05	no
**ENSG00000238084.4_RP3-469D22.1**	RP3-469D22.1	0.87	3.85	0.05	no
**ENSG00000142539.9_CTD-2545M3.6**	CTD-2545M3.6	2.60	9.37	0.05	no
**ENSG00000265558.1_MIR3918**	MIR3918	3.20	0.29	0.05	no
**ENSG00000263644.1_RP11-269G24.3**	RP11-269G24.3	0.01	0.90	0.05	no
**ENSG00000149635.2_OCSTAMP**	OCSTAMP	0.44	2.12	0.05	no
**ENSG00000256407.1_RP11-446E24.4**	RP11-446E24.4	0.33	1.61	0.05	no
**ENSG00000271225.1_BNIP3P4**	BNIP3P4	0.07	1.08	0.04	no
**ENSG00000254528.7_RP11-728F11.4**	RP11-728F11.4	0.51	2.18	0.04	no
**ENSG00000257746.1_RP11-202G11.2**	RP11-202G11.2	9.04	5.55	0.04	no
**ENSG00000281318.1_AC084854.1**	AC084854.1	1.66	0.16	0.04	no
**ENSG00000211650.2_IGLV5-45**	IGLV5-45	4.77	1.39	0.04	no
**ENSG00000277485.1_AL583842.4**	AL583842.4	0.91	3.37	0.03	no
**ENSG00000281990.1_CH17-212P11.5**	CH17-212P11.5	4.01	0.98	0.03	no
**ENSG00000211611.2_IGKV6-21**	IGKV6-21	5.16	1.73	0.03	no
**ENSG00000276775.1_IGHV4-4**	IGHV4-4	10.51	3.88	0.03	no
**ENSG00000240828.1_RPL21P4**	RPL21P4	0.14	1.36	0.03	no
**ENSG00000175643.8_RMI2**	RMI2	11.39	6.61	0.03	no
**ENSG00000274553.1_AC138035.3**	AC138035.3	0.03	0.68	0.03	no
**ENSG00000200418.1_SNORA63**	SNORA63	5.95	1.41	0.03	no
**ENSG00000269888.1_RP11-3P17.5**	RP11-3P17.5	26.05	105.75	0.03	no
**ENSG00000281808.1_SNORA17**	SNORA17	3.55	0.94	0.03	no
**ENSG00000167210.16_LOXHD1**	LOXHD1	1.49	7.78	0.02	no
**ENSG00000225415.2_RP3-509I19.1**	RP3-509I19.1	1.13	4.61	0.02	no
**ENSG00000273333.2_XXbac-BPG300A18.13**	XXbac-BPG300A18.13	1.77	0.30	0.02	no
**ENSG00000281044.1_AC022098.2**	AC022098.2	0.07	1.02	0.02	no
**ENSG00000224846.2_RP1-90J20.8**	RP1-90J20.8	0.29	1.62	0.02	no
**ENSG00000268199.2_CTD-3137H5.1**	CTD-3137H5.1	0.30	1.50	0.02	no
**ENSG00000265737.1_RP11-1157N2__B.2**	RP11-1157N2	1.99	0.59	0.02	no
**ENSG00000206630.1_SNORD60**	SNORD60	1.84	0.61	0.02	no
**ENSG00000229939.1_RP11-111F16.2**	RP11-111F16.2	4.14	15.90	0.02	no
**ENSG00000188394.6_GPR21**	GPR21	3.47	1.05	0.02	no
**ENSG00000240963.1_RP11-518L10.5**	RP11-518L10.5	1.15	3.01	0.02	no
**ENSG00000268083.5_AC104534.3**	AC104534.3	0.72	4.40	0.02	no
**ENSG00000253366.3_RP11-589F5.4**	RP11-589F5.4	3.29	0.88	0.02	no
**ENSG00000267827.5_CTC-471J1.8**	CTC-471J1.8	1.60	6.28	0.02	no
**ENSG00000279868.2_RN7SL3**	RN7SL3	3.84	1.19	0.02	no
**ENSG00000280602.1_AL627171.1**	AL627171.1	3.84	1.19	0.02	no
**ENSG00000281893.1_AL139099.3**	AL139099.3	3.84	1.19	0.02	no
**ENSG00000262406.2_MMP12**	MMP12	1.20	4.24	0.02	no
**ENSG00000199466.1_Y_RNA**	Y	0.07	1.02	0.02	no

Table S2. Transcriptomics analysis of the mRNA from PBMCs in subjects who received the placebo between the two time-points. The differential expressed genes (DE) correnpond to non-coding RNA and did not presented a known function. It were presented the fisrt 100 genes of the obtained list after the analysis.

ENSEMBL	Gene	Pre-vaccination mean	Post-vaccination mean	PP	DE
**ENSG00000278774.1_U2**	U2	22.68	0.00	1.00	yes
**ENSG00000280496.1_SNORA43**	SNORA43	41.13	3.17	1.00	yes
**ENSG00000137970.7_RPL7P9**	RPL7P9	7.62	0.00	1.00	yes
**ENSG00000276924.1_FP236383.9**	FP236383.9	68.46	3.18	1.00	yes
**ENSG00000277145.1_AL592188.7**	AL592188.7	68.46	3.18	1.00	yes
**ENSG00000277765.1_AL353644.11**	AL353644.11	68.46	3.18	1.00	yes
**ENSG00000278047.1_FP671120.6**	FP671120.6	68.46	3.18	1.00	yes
**ENSG00000256349.1_CTD-3074O7.11**	CTD-3074O7.11	0.41	10.34	1.00	yes
**ENSG00000207524.1_RNU6-33P**	RNU6-33P	0.00	5.74	1.00	yes
**ENSG00000274611.3_TBC1D3**	TBC1D3	12.33	1.36	1.00	yes
**ENSG00000232133.1_IMPDH1P10**	IMPDH1P10	1.11	7.19	1.00	yes
**ENSG00000254893.5_AC113404.1**	AC113404.1	4.15	0.19	1.00	yes
**ENSG00000256966.6_RP11-613M10.8**	RP11-613M10.8	22.10	5.04	0.94	no
**ENSG00000274049.4_INO80B-WBP1**	INO80B-WBP1	1.23	7.09	0.93	no
**ENSG00000230202.1_RP11-632C17__A.1**	RP11-632C17	6.93	1.63	0.92	no
**ENSG00000272993.1_RP11-196G18.24**	RP11-196G18.24	0.26	3.07	0.90	no
**ENSG00000273213.1_RP5-998N21.10**	RP5-998N21.10	1.92	0.04	0.89	no
**ENSG00000232702.3_RP3-437C15.1**	RP3-437C15.1	4.43	0.57	0.88	no
**ENSG00000265967.1_AC100830.4**	AC100830.4	1.54	0.06	0.81	no
**ENSG00000108825.17_PTGES3L-AARSD1**	PTGES3L-AARSD1	5.51	0.85	0.75	no
**ENSG00000265746.1_KYNUP2**	KYNUP2	3.25	0.26	0.57	no
**ENSG00000238997.1_AC105052.1**	AC105052.1	0.10	1.76	0.55	no
**ENSG00000253645.1_CTD-2544N14.3**	CTD-2544N14.3	0.50	5.51	0.23	no
**ENSG00000223643.6_RP11-108M9.1**	RP11-108M9.1	2.79	0.81	0.18	no
**ENSG00000273514.1_FOXD4L6**	FOXD4L6	0.36	1.80	0.16	no
**ENSG00000281371.1_INE2**	INE2	3.45	0.57	0.14	no
**ENSG00000205105.6_COX17P1**	COX17P1	3.14	0.62	0.14	no
**ENSG00000258800.1_CTD-2302E22.2**	CTD-2302E22.2	4.85	1.36	0.14	no
**ENSG00000256034.1_RP11-707G14.1**	RP11-707G14.1	0.08	1.35	0.11	no
**ENSG00000274582.1_SNORA16A**	SNORA16A	9.45	3.76	0.11	no
**ENSG00000258367.2_RP11-536C10.3**	RP11-536C10.3	2.22	0.52	0.09	no
**ENSG00000274804.1_AL353644.3**	AL353644.3	14.77	3.18	0.09	no
**ENSG00000278436.1_AL592188.11**	AL592188.11	14.77	3.18	0.09	no
**ENSG00000276353.1_Metazoa_SRP**	Metazoa	3.36	7.20	0.08	no
**ENSG00000263218.2_CTD-2561B21.7**	CTD-2561B21.7	2.45	0.61	0.08	no
**ENSG00000111780.8_AL021546.6**	AL021546.6	7.93	2.40	0.07	no
**ENSG00000196862.9_RGPD4**	RGPD4	0.68	3.23	0.06	no
**ENSG00000232573.1_RPL3P4**	RPL3P4	0.06	1.15	0.04	no
**ENSG00000259006.1_RP11-566K11.4**	RP11-566K11.4	0.27	1.57	0.04	no
**ENSG00000236360.2_RP11-334A14.2**	RP11-334A14.2	4.68	1.57	0.04	no
**ENSG00000256040.2_PAPPA-AS1**	PAPPA-AS1	2.26	4.47	0.04	no
**ENSG00000235486.1_AC114755.7**	AC114755.7	0.18	1.32	0.03	no
**ENSG00000260755.1_RP11-403P17.3**	RP11-403P17.3	0.16	1.54	0.03	no
**ENSG00000250902.1_SMAD1-AS1**	SMAD1-AS1	1.41	0.22	0.03	no
**ENSG00000274452.1_U2**	U2	24.34	87.36	0.03	no
**ENSG00000114349.9_GNAT1**	GNAT1	0.09	1.05	0.02	no
**ENSG00000200312.1_RN7SKP255**	RN7SKP255	7.02	3.35	0.02	no
**ENSG00000257839.1_RP11-290L1.2**	RP11-290L1.2	0.94	0.03	0.02	no
**ENSG00000279800.2_ABBA01017803.1**	ABBA01017803.1	0.74	0.04	0.02	no

**ENSG00000267105.1_CTD-2369P2.4**	CTD-2369P2.4	2.43	5.01	0.02	no
**ENSG00000269378.1_ITGB1P1**	ITGB1P1	3.15	9.98	0.02	no
**ENSG00000254741.1_RP11-661A12.7**	RP11-661A12.7	2.28	0.45	0.02	no
**ENSG00000179813.6_FAM216B**	FAM216B	18.47	6.14	0.02	no
**ENSG00000178568.13_ERBB4**	ERBB4	0.08	0.93	0.02	no
**ENSG00000232772.1_MTND4P22**	MTND4P22	2.22	0.63	0.02	no
**ENSG00000108702.3_CCL1**	CCL1	4.66	1.14	0.02	no
**ENSG00000172986.12_GXYLT2**	GXYLT2	11.58	16.62	0.02	no
**ENSG00000151790.8_TDO2**	TDO2	35.63	16.91	0.02	no
**ENSG00000197149.5_RP11-452G18.2**	RP11-452G18.2	2.75	1.02	0.02	no
**ENSG00000272593.1_RP11-339B21.11**	RP11-339B21.11	0.36	1.38	0.01	no
**ENSG00000268810.1_AC007193.9**	AC007193.9	0.10	1.10	0.01	no
**ENSG00000233653.3_CICP7**	CICP7	0.14	1.36	0.01	no
**ENSG00000136696.10_IL36B**	IL36B	5.56	1.92	0.01	no
**ENSG00000263412.1_RP5-890E16.2**	RP5-890E16.2	14.32	19.86	0.01	no
**ENSG00000181374.7_CCL13**	CCL13	16.98	6.15	0.01	no
**ENSG00000217576.7_RP11-248G5.8**	RP11-248G5.8	0.47	1.73	0.01	no
**ENSG00000197358.9_BNIP3P1**	BNIP3P1	0.12	1.24	0.01	no
**ENSG00000266486.1_FAM106CP**	FAM106CP	0.66	0.07	0.01	no
**ENSG00000271153.1_RPL23AP88**	RPL23AP88	0.98	2.94	0.01	no
**ENSG00000237568.1_RP4-620F22.2**	RP4-620F22.2	5.65	1.06	0.01	no
**ENSG00000262079.1_RP11-515O17.3**	RP11-515O17.3	7.68	11.75	0.01	no
**ENSG00000261499.2_CH17-260O16.1**	CH17-260O16.1	4.05	0.84	0.01	no
**ENSG00000273184.1_RP11-212P7.3**	RP11-212P7.3	11.19	4.60	0.01	no
**ENSG00000214195.4_HMGN2P31**	HMGN2P31	0.06	0.68	0.01	no
**ENSG00000200830.1_RN7SKP134**	RN7SKP134	0.83	0.05	0.01	no
**ENSG00000250983.1_RP11-91K8.2**	RP11-91K8.2	0.21	1.19	0.01	no
**ENSG00000276101.1_RP11-455O6.8**	RP11-455O6.8	0.60	0.11	0.01	no
**ENSG00000231542.1_TAB3-AS1**	TAB3-AS1	2.82	0.64	0.01	no
**ENSG00000281295.1_SNORA50**	SNORA50	0.69	3.34	0.01	no
**ENSG00000261915.6_RP11-542C16.2**	RP11-542C16.2	6.21	13.68	0.01	no
**ENSG00000269583.1_L34079.4**	L34079.4	0.16	1.24	0.01	no
**ENSG00000248871.1_TNFSF12-TNFSF13**	TNFSF12-TNFSF13	25.19	11.54	0.01	no
**ENSG00000276418.4_RP11-26J3.4**	RP11-26J3.4	1.07	2.63	0.01	no
**ENSG00000273628.1_RP11-756A22.7**	RP11-756A22.7	6.52	3.12	0.01	no
**ENSG00000147896.3_IFNK**	IFNK	20.46	14.35	0.01	no
**ENSG00000234664.1_HMGN2P5**	HMGN2P5	19.81	9.24	0.01	no
**ENSG00000187559.5_FOXD4L3**	FOXD4L3	1.40	0.39	0.01	no
**ENSG00000224821.5_COL4A2-AS2**	COL4A2-AS2	2.71	0.99	0.01	no
**ENSG00000253082.1_AC007365.2**	AC007365.2	0.12	1.00	0.01	no
**ENSG00000269941.1_RP5-1172N10.4**	RP5-1172N10.4	0.93	2.09	0.01	no
**ENSG00000261427.6_CTD-2349B8.1**	CTD-2349B8.1	0.56	0.12	0.01	no
**ENSG00000136842.13_TMOD1**	TMOD1	31.97	19.37	0.01	no
**ENSG00000225341.1_AC013269.4**	AC013269.4	0.08	0.68	0.01	no
**ENSG00000215692.2_AC114730.8**	AC114730.8	1.76	0.32	0.01	no
**ENSG00000273667.1_MIR142**	MIR142	0.17	1.00	0.01	no
**ENSG00000104967.6_NOVA2**	NOVA2	5.33	8.98	0.01	no
**ENSG00000261779.1_RP11-69H7.3**	RP11-69H7.3	0.09	0.74	0.01	no
**ENSG00000274985.1_PTCHD3P1**	PTCHD3P1	0.27	1.08	0.01	no
**ENSG00000221837.5_KRTAP10-9**	KRTAP10-9	0.08	0.53	0.01	no

Table S3. List of the 66 genes differentially expressed based on the threshold level (fold change (log2) ≥ 1 and P-value < 0.05. It is presented the Ensembl genecode, the gene symbol, the means after gene quantification in non-responder and responder groups, the Fold Change (FC) and the log2 FC between group, respectively.

ENSEMBL	Gene symbol	No-Responder Mean	Responder Mean	FC	log2FC
**ENSG00000137970.7_RPL7P9**	RPL7P9	0.000	6.123	-	-
**ENSG00000229344.1_RP5-857K21.7**	RP5-857K21.7	28.906	0.212	0.007	-7.092
**ENSG00000230202.1_RP11-632C17__A.1**	RP11-632C17	24.257	0.488	0.020	-5.636
**ENSG00000230629.2_RPS23P8**	RPS23P8	20.111	0.329	0.016	-5.935
**ENSG00000250182.3_EEF1A1P13**	EEF1A1P13	70.143	2.296	0.033	-4.933
**ENSG00000278774.1_U2**	U2	13.253	0.000	-	-
**ENSG00000281220.1_AC011404.1**	AC011404.1	5.094	51.739	10.157	3.344
**ENSG00000213300.5_HNRNPA3P6**	HNRNPA3P6	7.900	0.560	0.071	-3.819
**ENSG00000233870.1_AC007881.4**	AC007881.4	3.743	0.050	0.013	-6.233
**ENSG00000257000.1_RP13-820C6.2**	RP13-820C6.2	0.147	3.575	24.308	4.603
**ENSG00000237568.1_RP4-620F22.2**	RP4-620F22.2	0.027	4.823	175.747	7.457
**ENSG00000259032.2_ENSAP2**	ENSAP2	0.683	7.199	10.539	3.398
**ENSG00000235558.3_RP11-1198D22.1**	RP11-1198D22.1	0.000	1.814	-	-
**ENSG00000232133.1_IMPDH1P10**	IMPDH1P10	1.149	5.469	4.760	2.251
**ENSG00000254452.1_RP11-867G23.4**	RP11-867G23.4	0.001	1.939	3026.670	11.564
**ENSG00000244734.3_HBB**	HBB	2776.480	462.308	0.167	-2.586
**ENSG00000281990.1_CH17-212P11.5**	CH17-212P11.5	3.041	0.237	0.078	-3.681
**ENSG00000230395.1_AC092651.1**	AC092651.1	0.051	1.970	38.338	5.261
**ENSG00000207322.1_RNU1-89P**	RNU1-89P	0.056	1.350	24.100	4.591
**ENSG00000137959.15_IFI44L**	IFI44L	432.484	1569.355	3.629	1.859
**ENSG00000158578.18_ALAS2**	ALAS2	69.313	11.780	0.170	-2.557
**ENSG00000206172.8_HBA1**	HBA1	461.389	69.407	0.150	-2.733
**ENSG00000211696.2_TRGV8**	TRGV8	10.522	36.526	3.471	1.795
**ENSG00000281371.1_INE2**	INE2	4.422	0.526	0.119	-3.071
**ENSG00000134321.11_RSAD2**	RSAD2	334.218	1103.978	3.303	1.724
**ENSG00000102854.14_MSLN**	MSLN	0.347	2.606	7.509	2.909
**ENSG00000185641.6_CTD-2287O16.1**	CTD-2287O16.1	10.711	3.954	0.369	-1.438
**ENSG00000158497.2_HMHB1**	HMHB1	0.976	0.085	0.087	-3.517
**ENSG00000238749.1_Y_RNA**	Y	7.493	1.172	0.156	-2.676
**ENSG00000238933.1_Y_RNA**	Y	7.493	1.172	0.156	-2.676
**ENSG00000275904.1_Y_RNA**	Y	7.493	1.172	0.156	-2.676
**ENSG00000262429.1_RP5-1050D4.3**	RP5-1050D4.3	0.089	1.636	18.295	4.193
**ENSG00000268614.1_CTD-2207O23.10**	CTD-2207O23.10	0.146	2.304	15.797	3.982
**ENSG00000268565.1_AC005339.2**	AC005339.2	0.632	3.537	5.600	2.485
**ENSG00000207524.1_RNU6-33P**	RNU6-33P	0.663	4.113	6.207	2.634
**ENSG00000279364.1_RP11-463I20.1**	RP11-463I20.1	0.000	2.327	-	-
**ENSG00000253838.1_RP11-44K6.2**	RP11-44K6.2	1.457	6.106	4.192	2.068
**ENSG00000234851.4_RPL23AP42**	RPL23AP42	74.075	12.612	0.170	-2.554
**ENSG00000276924.1_FP236383.9**	FP236383.9	33.811	5.168	0.153	-2.710
**ENSG00000277145.1_AL592188.7**	AL592188.7	33.811	5.168	0.153	-2.710
**ENSG00000277765.1_AL353644.11**	AL353644.11	33.811	5.168	0.153	-2.710
**ENSG00000278047.1_FP671120.6**	FP671120.6	33.811	5.168	0.153	-2.710
**ENSG00000204538.3_PSORS1C2**	PSORS1C2	0.152	1.567	10.337	3.370
**ENSG00000265558.1_MIR3918**	MIR3918	0.053	2.192	41.695	5.382
**ENSG00000273443.1_RP11-54O7.18**	RP11-54O7.18	18.787	9.348	0.498	-1.007
**ENSG00000259684.1_RP11-120K9.2**	RP11-120K9.2	3.493	20.511	5.872	2.554
**ENSG00000280133.1_RP4-789D17.3**	RP4-789D17.3	1.092	0.000	-	-
**ENSG00000196611.4_MMP1**	MMP1	45.444	9.773	0.215	-2.217
**ENSG00000250562.1_RPL38P4**	RPL38P4	6.212	1.685	0.271	-1.882
**ENSG00000265590.9_AP000275.65**	AP000275.65	7.213	1.444	0.200	-2.321
**ENSG00000219928.2_RP11-40C6.2**	RP11-40C6.2	10.251	3.732	0.364	-1.458

**ENSG00000126709.14_IFI6**	IFI6	343.618	974.326	2.835	1.504
**ENSG00000269349.1_CTD-3099C6.5**	CTD-3099C6.5	3.736	0.639	0.171	-2.547
**ENSG00000273124.1_RP11-236B18.5**	RP11-236B18.5	1.609	5.319	3.305	1.725
**ENSG00000253683.1_CTB-79E8.3**	CTB-79E8.3	2.947	0.445	0.151	-2.728
**ENSG00000274527.4_RP11-146B14.1**	RP11-146B14.1	0.243	1.495	6.156	2.622
**ENSG00000260349.1_RP11-473I1.5**	RP11-473I1.5	4.025	2.017	0.501	-0.997
**ENSG00000274049.4_INO80B-WBP1**	INO80B-WBP1	5.336	1.396	0.262	-1.935
**ENSG00000176868.2_RP11-334J6.7**	RP11-334J6.7	1.452	6.859	4.723	2.240
**ENSG00000139574.8_NPFF**	NPFF	0.785	3.627	4.622	2.209
**ENSG00000169877.9_AHSP**	AHSP	2.517	0.415	0.165	-2.599
**ENSG00000267062.1_CTD-2659N19.10**	CTD-2659N19.10	0.098	1.831	18.608	4.218
**ENSG00000165949.12_IFI27**	IFI27	18.095	46.820	2.587	1.372
**ENSG00000226004.1_RP11-10J5.1**	RP11-10J5.1	2.358	6.673	2.830	1.501
**ENSG00000166670.9_MMP10**	MMP10	5.275	1.001	0.190	-2.398
**ENSG00000204850.4_CTB-158D10.3**	CTB-158D10.3	2.785	0.673	0.242	-2.049
**ENSG00000228695.9_CES1P1**	CES1P1	2.986	0.524	0.175	-2.512
**ENSG00000277745.1_H2AFB3**	H2AFB3	1.867	0.313	0.168	-2.575
**ENSG00000236852.1_RP11-3D23.1**	RP11-3D23.1	1.381	0.078	0.056	-4.152
**ENSG00000185261.13_KIAA0825**	KIAA0825	83.668	113.638	1.358	0.442
**ENSG00000280496.1_SNORA43**	SNORA43	13.094	54.846	4.189	2.067
**ENSG00000278599.4_TBC1D3E**	TBC1D3E	5.594	21.364	3.819	1.933
**ENSG00000276612.3_CH507-9B2.4**	CH507-9B2.4	4.633	1.363	0.294	-1.765
**ENSG00000225964.5_NRIR**	NRIR	7.802	18.087	2.318	1.213

Table S4. List of the 53 pathways obtained after the gene enrichment analysis. It was acquired using the KOBAS software and the Gene Ontology (GO) database.

**TERM**	**ID**	**INPUT**	**TOTAL**	**p-value**	**q-value**
**hemoglobin complex**	GO:0005833	3	11	2.20E+04	3.74E+06
**hemoglobin binding**	GO:0030492	2	5	1.58E+06	0.00119916239106
**defense response to virus**	GO:0051607	4	201	2.98E+06	0.00119916239106
**type I interferon signaling pathway**	GO:0060337	3	67	3.21E+06	0.00119916239106
**haptoglobin binding**	GO:0031720	2	9	4.12E+06	0.00119916239106
**organic acid binding**	GO:0043177	2	10	4.94E+06	0.00119916239106
**haptoglobin-hemoglobin complex**	GO:0031838	2	10	4.94E+06	0.00119916239106
**oxygen carrier activity**	GO:0005344	2	13	7.84E+05	0.00166658786227
**oxygen transport**	GO:0015671	2	14	8.96E+04	0.00169212817493
**endocytic vesicle lumen**	GO:0071682	2	15	0.000101472914417	0.00172503954509
**hydrogen peroxide catabolic process**	GO:0042744	2	27	0.000300971582653	0.00465137900464
**peroxidase activity**	GO:0004601	2	31	0.000390567783959	0.00542371308726
**oxygen binding**	GO:0019825	2	32	0.000414754530202	0.00542371308726
**positive regulation of cell death**	GO:0010942	2	34	0.000465265037048	0.00564964687843
**extracellular region**	GO:0005576	7	1746	0.000595606833788	0.00675021078293

**bicarbonate transport**	GO:0015701	2	41	0.000664367652023	0.00695629095313
**collagen catabolic process**	GO:0030574	2	42	0.000695629095313	0.00695629095313
**response to hydrogen peroxide**	GO:0042542	2	48	0.000897879646719	0.00803365999696
**erythrocyte differentiation**	GO:0030218	2	48	0.000897879646719	0.00803365999696
**extracellular space**	GO:0005615	6	1446	0.00134798404106	0.011457864349
**apoptotic signaling pathway**	GO:0097190	2	64	0.00155871048229	0.01261813
**extracellular matrix disassembly**	GO:0022617	2	66	0.00165357562207	0.0127776298069
**cellular oxidant detoxification**	GO:0098869	2	75	0.00211368342146	0.015622877463
**molecular_function**	GO:0003674	4	717	0.00344146657523	0.0243770549079
**receptor-mediated endocytosis**	GO:0006898	2	102	0.00381326123018	0.0259301763652
**metalloendopeptidase activity**	GO:0004222	2	106	0.00410486944324	0.0262904388019
**blood microparticle**	GO:0072562	2	109	0.00433018992031	0.0262904388019
**mitochondrial membrane**	GO:0031966	2	109	0.00433018992031	0.0262904388019
**mitochondrial inner membrane**	GO:0005743	3	382	0.00460977595517	0.0270228245648
**hemoglobin alpha binding**	GO:0031721	1	5	0.00530931381161	0.0291155918701
**hemoglobin metabolic process**	GO:0020027	1	5	0.00530931381161	0.0291155918701
**cellular_component**	GO:0005575	3	413	0.0057157236667	0.0303647819794
**hemoglobin biosynthetic process**	GO:0042541	1	6	0.00619157387803	0.0309578693901
**renal absorption**	GO:0070293	1	6	0.00619157387803	0.0309578693901
**heme binding**	GO:0020037	2	136	0.00660830307265	0.0320974720671
**positive regulation of T-helper 2 cell cytokine production**	GO:2000553	1	7	0.00707308540139	0.0321941506655
**negative regulation of mitochondrial depolarization**	GO:0051902	1	7	0.00707308540139	0.0321941506655
**viral process**	GO:0016032	3	452	0.00731706261242	0.0321941506655
**regulation of membrane depolarization**	GO:0003254	1	8	0.00795384898794	0.0321941506655
**acute inflammatory response to antigenic stimulus**	GO:0002438	1	8	0.00795384898794	0.0321941506655
**regulation of protein export from nucleus**	GO:0046825	1	8	0.00795384898794	0.0321941506655
**sterol esterase activity**	GO:0004771	1	8	0.00795384898794	0.0321941506655
**oxygen homeostasis**	GO:0032364	1	9	0.00883386524347	0.0341308429861

**protoporphyrinogen IX biosynthetic process**	GO:0006782	1	9	0.00883386524347	0.0341308429861
**negative regulation of heart rate**	GO:0010459	1	10	0.00971313477328	0.0345675084947
**negative regulation of appetite**	GO:0032099	1	10	0.00971313477328	0.0345675084947
**regulation of epidermal growth factor receptor signaling pathway**	GO:0042058	1	10	0.00971313477328	0.0345675084947
**mitochondrial outer membrane**	GO:0005741	2	167	0.00976023769262	0.0345675084947
**positive regulation of immune response**	GO:0050778	1	12	0.0114694360747	0.0397919210754
**cellular protein metabolic process**	GO:0044267	2	198	0.0134564607739	0.0457519666312
**nuclear nucleosome**	GO:0000788	1	15	0.0140983026899	0.0460906049477
**glycine binding**	GO:0016594	1	15	0.0140983026899	0.0460906049477
**lamin binding**	GO:0005521	1	16	0.0149731045509	0.0480269391254
